# Radiolabeling polymeric micelles for in vivo evaluation: a novel, fast, and facile method

**DOI:** 10.1186/s13550-016-0167-x

**Published:** 2016-02-09

**Authors:** Adrianus C. Laan, Costanza Santini, Laurence Jennings, Marion de Jong, Monique R. Bernsen, Antonia G. Denkova

**Affiliations:** Department of Radiation Science and Technology, Delft University of Technology, Mekelweg 15, 2629 JB Delft, The Netherlands; Department of Radiology, Erasmus Medical Center, Dr. Molewaterplein 50-60, 3015 GE Rotterdam, The Netherlands; Institut Charles Sadron (UPR22-CNRS), 23 Rue du Loess, 67034 Strasbourg Cedex 2, France

**Keywords:** Radiolabeling, Polymeric micelles, Amphiphilic diblock copolymers, SPECT

## Abstract

**Background:**

Single photon emission computed tomography (SPECT) is an indispensable tool in the determination of the in vivo fate of polymeric micelles. However, for this purpose, the micelles need to be radiolabeled, and almost all radiolabeling procedures published to date involve the conjugation of a chelating agent to the constituting polymer, which could actually affect their biodistribution. In this paper, we report a new facile method for radiolabeling polystyrene-b-poly(ethylene oxide) diblock copolymer micelles without the necessity of any chemical modification. Instead, we entrap the radiolabel (i.e., ^111^In) in the micellar core during the formation of the micelles by using tropolone as lipophilic ligand.

**Methods:**

Micelles were prepared by emulsifying a polymer solution in chloroform with a buffer containing ^111^In and lipophilic ligand tropolone, by stirring for about 2 h. The produced micelles were physically characterized by means of dynamic light scattering and transmission electron microscopy. The biological properties of the radiolabeled micelles were determined by means of in vivo and ex vivo evaluation. SPECT analysis was done on Balb/c-nu mice, after administration of 1 mg micelles containing 22 MBq of ^111^In. SPECT images were obtained over 24 h. Biodistribution of the micelles was assessed also ex vivo.

**Results:**

The radiolabeling method is robust and reproducible with constant radiolabeling efficiency (~30 %) even at indium concentrations that are much higher than the necessary for in vivo studies, and the radiolabel retention is more than 80 % in mouse serum at 48 h. Radiolabeled micelles having hydrodynamic radius of 97 ± 13 nm have been successfully evaluated in vivo and ex vivo in non-tumor-bearing mice, revealing significant blood circulation up to at least 24 h post injection, with low accumulation in most organs except for the liver and spleen, which are the natural organs for clearance of nanoparticles.

**Conclusions:**

An easy and robust radiolabeling method has been developed, and its applicability is demonstrated in animal studies, showing its value for future investigation of polymeric micelles as nanocarriers in tumor-bearing mice.

**Electronic supplementary material:**

The online version of this article (doi:10.1186/s13550-016-0167-x) contains supplementary material, which is available to authorized users.

## Background

Polymeric micelles are promising nanocarriers for pharmaceutical formulations due to their ability to encapsulate hydrophobic substances, overcoming in this way the poor solubility of some drugs and allowing transport through the blood stream to the site of interest [1, 2]. These micelles are constituted by amphiphilic block copolymers, which in aqueous solutions are able to self-assemble into supramolecular structures. In contrast to self-assemblies composed of low molecular weight surfactants, diblock copolymer micellar aggregates usually exhibit a low critical micelle concentration (CMC) which enhances their integrity even at low concentration [3]. By varying the composition, the size, and the structure of the block copolymers, i.e., tuning the hydrophilic and hydrophobic block ratio, and/or by adjusting the micelle production method, it is possible to design polymeric micelles with well-defined dimensions and morphologies [4, 5]. These favorable characteristics of polymeric micelles have already led to clinical applications for systemic chemotherapy [6, 7]. In these applications, bringing the micelles to the desired site is essential for therapeutic success, which can be accomplished by exploiting either active or passive targeting pathways. Nanocarriers in general can accumulate in diseased tissue due to the enhanced permeability and retention (EPR) effect, also referred to as passive targeting. Growing tissue, especially tumors, can establish new vascular systems to supply themselves with oxygen and nutrients. The newly formed vessels, however, have a discontinuous endothelium and lack of lymphatic drainage, enhancing in this way extravasation and retention of large particles over time [8, 9]. Long blood circulation time can increase the needed in vivo availability and is therefore an important factor to improve accumulation in tumor tissue. In active targeting, antibodies or peptides are used to ensure tumor uptake, but in the case of nanoparticles even when such targeting vectors are present, it is still imperative that these entities remain in blood circulation for sufficiently long time [10].

Even though the scientific community is gaining considerable knowledge about the various factors that can affect the biodistribution of nanoparticles, it is still difficult to predict the in vivo behavior of new formulations, making pre-clinical evaluation absolutely essential. Nuclear imaging approaches such as SPECT (single photon emission computed tomography) and PET (positron emission tomography) are very suitable for this purpose, since they offer high detection sensitivity at high temporal and spatial resolution. These techniques do, however, require radiolabeling of the micelles prior to imaging. Several methods for radiolabeling of polymeric micelles are described in the literature, all involving the conjugation of the micelles with a chelate or complexing agent (e.g., DOTA or DTPA) which requires additional synthetic steps [11]. In addition, the attachment of such a chelate could alter the corona of the micelles and hence their biodistribution and pharmacokinetics [12–14].

In this paper, we present a new, facile radiolabeling method based on passive loading that is applied to micelles composed of the diblock copolymer polystyrene-block-poly(ethylene oxide) (PS-b-PEO). The non-ionic hydrophilic corona of the micelles is composed of PEO, often referred to as poly(ethylene glycol), which is commonly used in nano-particle formulations to enhance their stealthy surface chemistry [2, 13]. The hydrophobic counterpart is formed by the PS, which has a high glass-transition temperature (*T*_g_) and is extremely hydrophobic, ensuring stability of the micelles and a low release rate of any hydrophobic load [14]. The radiolabeling method described here bypasses the covalent attachment of a chelating agent since a lipophilic ligand (tropolone) complexed with the radionuclide (in this case ^111^In) is entrapped in the micellar core leaving the PEO corona unaffected. In this paper, we describe this radiolabeling strategy and demonstrate the ease and efficiency of this procedure as well as an initial in vivo evaluation of these micelles in healthy mice using SPECT.

## Methods

### Chemicals

The block copolymer PS-b-PEO with a *M*_n_ (number average molar mass) of 9500-b-18,000 g/mol was purchased from Polymer Source (Quebec, Canada). The block copolymer was nearly monodisperse with a *M*_w_/*M*_n_ ratio (*M*_w_ is the weight average molar mass) of 1.09. The ^111^InCl_3_ was obtained as solution in 10 mM hydrochloric acid from Mallinckrodt Pharmaceuticals (Petten, The Netherlands) with a specific activity of 1.72 MBq/pmol. Indium chloride, Sephadex G-25 and Sepharose 4B size exclusion chromatography resins, 4-(2-hydroxyethyl)-1-piperazine ethanesulfonic acid (HEPES), and 1,4-piperazinediethanesulfonic acid sodium salt (PIPES) were purchased from Sigma Aldrich, and tropolone from Merck. Ultrapure water was prepared with the in-house Milli-Q system from Merck Millipore.

### Production and radiolabeling of micelles

Formation of ^111^In-tropolone complexes was executed by adding 50 kBq ^111^InCl_3_ to 2.3 mL of 10 mM HEPES buffer solution (pH 7.4) containing 0.8 mM of tropolone. The sample was incubated for about 5 min at room temperature. Subsequently, 100 μL of a solution of PS-b-PEO block copolymer in chloroform was added to reach a polymer concentration of 4.3 mg/mL. The mixture was stirred at room temperature in a fume hood using a glass stirring bar in an open glass vial for about 2 h until the chloroform had evaporated. Unencapsulated ^111^In-tropolone was removed from the radiolabeled micelles by means of size exclusion chromatography (SEC) using a Sephadex G-25 column with a diameter of 1 cm and a length of 30 ± 1 cm, using 10 mM HCl as eluent. The ^111^In activity in the eluted fractions was analyzed using a Wallac WIZARD^2^ 2480 Automatic Gamma Counter (PerkinElmer), by measuring the peak area of the 171- and 245-keV photon peaks. The labeling efficiency was determined as the amount of ^111^In encapsulated in the micelles relative to the total amount of ^111^In added to the sample.

### Physical characterization of the micelles

Samples used for the physical characterization of the micelles were prepared exactly the same way as described above but using non-radioactive indium chloride.

The sample used in the dynamic light scattering (DLS) studies was prepared in duplicate and contained 4.3 mg/mL PS-b-PEO. Prior to the measurements, the sample was diluted 10, 50, and 200 times using HEPES buffer and each diluted sample was measured three times following protocols described previously [15]. The DLS apparatus consisted of a JDS Uniphase 633 nm 35 mW laser, an ALV sp 125 s/w 93 goniometer, a fiber detector, and a PerkinElmer photon counter. An ALV-5000/epp correlator and software completed the set-up. The DLS sample cell was placed in a temperature-regulated bath containing toluene as the index-matching fluid. The intensity autocorrelation function, *g*^(2*)*^(*τ*), was determined at 90°. The measurements were performed at 22 ± 1 °C. The *R*_H_ of the micelles was determined using the CONTIN method, and the radius was calculated from the diffusion coefficient of the particles using the Einstein-Stokes equation.

The samples used in the transmission electron microscopy (TEM) analysis contained 1.1 mg/mL and 4.3 mg/mL PS-b-PEO. Prior to analysis, a five-time dilution was made in HEPES buffer. A drop of the micellar solution was pipetted onto a carbon-coated copper grid of 200 mesh (Quantifoil, Jena, Germany). Excess liquid was removed with a tissue, and the grid was left to dry before placing the specimen into the microscope. A JEM 1400 TEM (JEOL) was used with a LaB_6_ electron source, operated at an acceleration voltage of 120 keV.

### Partition studies

For the partition study, 4 mL of either PIPES or HEPES buffer, containing 0.8 μM tropolone and 5 kBq ^111^In was mixed with an equal volume of chloroform during 30 s using a vortex. The aqueous and organic phases were left to separate gravimetrically, after which 1 mL of each phase was transferred into a counting vial and measured using the automatic gamma counter described previously. The distribution ratio (*D*) of the ^111^In was calculated according to the following equation:

1 $$ D=\frac{{\left[{}^{111}\mathrm{In}\right]}_{\mathrm{chloroform}}}{{\left[{}^{111}\mathrm{In}\right]}_{\mathrm{HEPES}\ \mathrm{buffer}}} $$

In which [^111^In]_chloroform_ is the concentration of ^111^In in the chloroform phase, and [^111^In]_HEPES_ buffer is the concentration of ^111^In in the aqueous phase. The *D* was assessed at pH 4.5, 5.5, 6.5, 7.4, and 8.5.

### Optimization of the radiolabeling parameters

In the optimization studies, the following parameters were sequentially tested: the polymer concentration, the ^111^In activity (i.e., the amount of indium), the tropolone concentration, and the pH. The polymer concentration varied from 0.22 to 8.7 mg/mL. In the optimization of the indium activity studies, non-radioactive indium was used in addition to ^111^In for safety reasons. The indium concentration ranged from 1.3 pM to 130 nM, corresponding to 5 kBq and 500 MBq ^111^In per sample of 2.3 mL, respectively, which is referred to as ^111^In equivalent. Stock solutions of non-radioactive InCl_3_ were prepared in 10 mM HCl, and for quantification, either 5 or 50 kBq of ^111^In was added to the sample. The tropolone concentration was varied from 0.8 nM to 80 μM, using stock solutions of tropolone in HEPES. PIPES buffers at pH 4.5, 5.5, and 6.5 and HEPES buffers at pH 7.4 and 8.5 were used in the pH optimization studies.

### Radiolabeling of preformed micelles

The micelles were prepared as follows: 100 μL of a PS-b-PEO block copolymer solution in chloroform was added to 2.3 mL of HEPES buffer containing 0.8 mM tropolone. The mixture was stirred until the chloroform had evaporated. To this solution, ^111^In-tropolone chloroform solution was added in portions of 2 μL, which was itself prepared by extracting the ^111^In into chloroform from a solution containing 1 mM tropolone in HEPES. The addition was done at 30-s intervals, until an activity of 50 kBq was reached. The unencapsulated ^111^In-tropolone was removed from the radiolabeled micelles by means of SEC. The ^111^In activity was measured as previously described using an automated gamma counter. Labeling efficiency was calculated relative to the total amount of ^111^In added to the sample.

### Retention studies

The retention of the ^111^In radiolabel in the micelles was assessed in PBS and in mouse serum. For both samples, 3 mL of the radiolabeled micelles solution was mixed with an equal volume of either PBS or serum. As control, 1 mL of the same sample was analyzed on the day of preparation. The samples were stored in an incubator at 37 °C, and after 24 and 48 h, the retention of the radiolabel was analyzed. The micelles in the serum samples were separated from the released ^111^In using a Sepharose 4B gel SEC column with a diameter of 1 cm and a length of 30 ± 1 cm. The micelles in the PBS were separated from the released ^111^In by using a Sephadex G-25 SEC column having the same dimensions as in the serum studies, using 10 mM HCl as eluent.

### In vivo and ex vivo evaluation

Micelles for in vivo tests were prepared as described in the “Production and radiolabeling of micelles” section, using a starting concentration of polymer adjusted to the number of animals and the required final polymer amount of 1 mg per animal. The sample was centrifuged for 90 min at 2300 g using Amicon® Ultra-4 centrifugal filter devices (Merck Millipore). The concentrated micellar solution was recovered from the filter and filled up to the required injection volume with PBS.

The biodistribution of PS-b-PEO micelles was tested in Balb/c-nu mice (Janvier Labs, Le Genest-Saint-Isle, France). Three mice received an injection of 200 μL of solution containing 22 MBq of ^111^In in 1 mg of micelles. Immediately after the injection, the mice were anesthetized with a gas mixture of isoflurane (Pharmachemie, Haarlem, The Netherlands) (4 % induction, 2 % maintenance) and oxygen (0.8 %) and subjected to SPECT/CT scan (NanoSPECT/CT, Bioscan Inc., California, USA). One mouse, randomly chosen, was subjected to a dynamic scan during the first 60 min post injection (pi) using a matrix of 256 × 256 pixels, 16 projections, 20 s per projection. Static images were further acquired 30 min pi, 4 h pi, and 24 h pi, using a matrix of 256 × 256 pixels, 20 projections with 60 s per projection. A CT scan (240 projections, 500 ms exposure time 55 kVp tube voltage) was performed for anatomical reference.

For biodistribution analyses after the last SPECT/CT acquisition, the mice were euthanized by cervical dislocation. Blood was immediately drawn via heart puncture and stored in a collection vial, then both the cadaver and the vial containing the collected blood were measured in a dose calibrator (Comecer VDC-404, COMECER Netherlands, Joure, The Netherlands) to have a measure of retention of radioactivity in the body. After the total body count, a selection of different organs was harvested. The uptake of the micelles in different tissues was calculated using the Wallac Wizard 1480 automated gamma counter (PerkinElmer) by measuring the emitted radiation. Part of the spleen, part of the liver, and the complete right kidney of one mouse were collected and stored at −80 °C for ex vivo autoradiography evaluation. From the frozen tissues, 15-μm tissue sections were cut, mounted on glasses, and processed for ex vivo autoradiography.

The slides were placed in a sensitive phosphor-imaging screen (PerkinElmer), and read out was performed 3 days later using the Cyclone Storage Phosphor System (Packard). Quantification of the autoradiograms was carried out with Optiquant (Software version 5.0. PerkinElmer).

All animal experiments were performed in accordance with the Dutch animal welfare regulations and approved by the Central Animal Testing Committee (Dutch: Centrale Commissie Dierproeven).

### Data analysis

The obtained in vitro data were processed with Microsoft Excel (Microsoft Office Professional Plus 2010 package). ANOVA was used for statistical evaluation. All the in vivo data collected were analyzed with GraphPad Prism (GraphPad Software, version 5).

## Results

### Physical characterization of the micelles

**Fig. 1:**
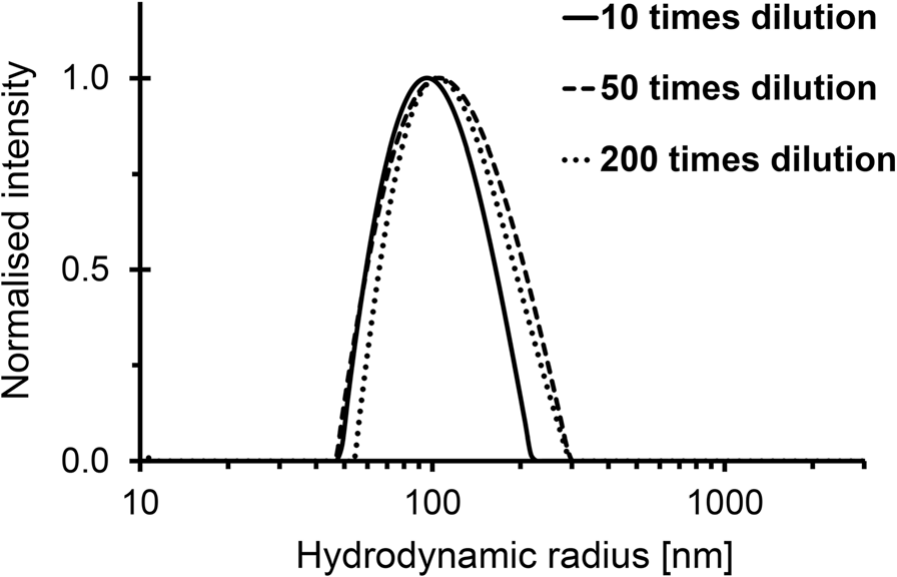
Size distribution of the PS-b-PEO micelles. Micelles are prepared at a polymer concentration of 4.3 mg/mL. The hydrodynamic radius has been determined by DLS for samples that have been diluted at 10, 50, and 200 times

The morphology of the micelles has been assessed by DLS and TEM. Figure 1 shows the *R*_H_ of the micelles for different dilutions. According to the DLS analysis, the micelles appear to have good stability upon dilution, having variations in the hydrodynamic radius within the measurement uncertainty. The overall average hydrodynamic radius has been found to be 97 ± 13 nm (*N* = 18).

**Fig. 2:**
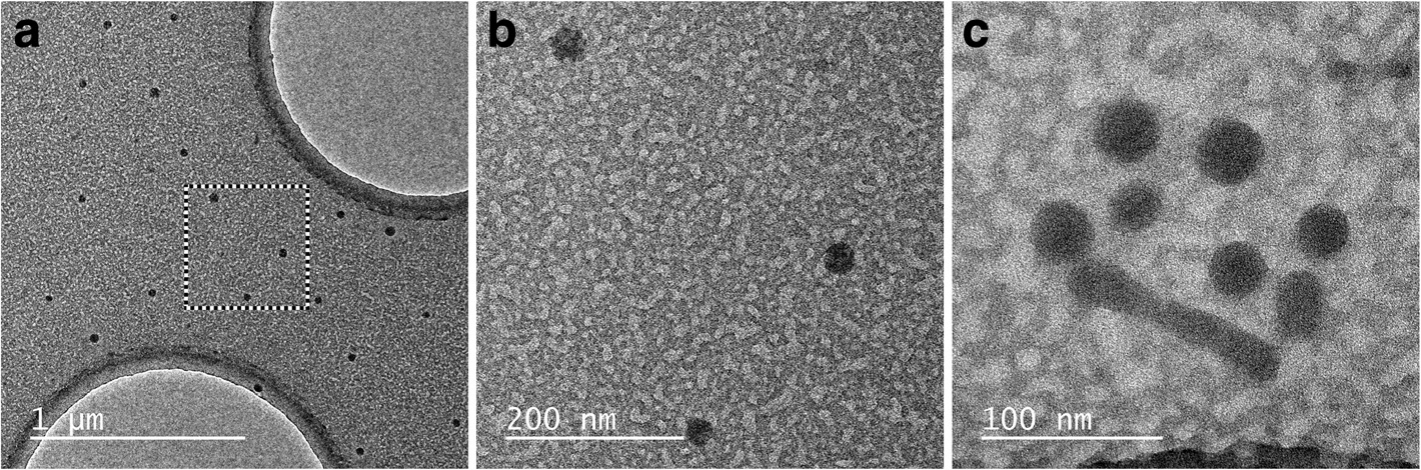
TEM images of PS-b-PEO micelles. Images of **a** spherical micelles, **b** magnified image of a **c** rod-like micelle between a few spherical ones. The concentration of the samples in **a** and **b** is 4.3 mg/mL and in **c** is 1.1 mg/mL

Three representative TEM images of the micelles are given in Fig. 2, in which the PS core is clearly visible as dark stains on a grey background. The PEO corona has lower electron density compared to the PS, and hence, it is (nearly) invisible. Most of the micelles appear to have spherical shape with diameter of the PS core ranging from 25 to 40 nm, irrespective of the polymer concentration. A small number of rod-like micelles with different lengths have also been observed (Fig. 2c). Their presence can explain the larger dimensions of the micelles measured by DLS since the presence of a small number of large objects can contribute significantly to the scattered light signal leading to a shift of the apparent hydrodynamic size to larger values.

### Radiolabeling

Prior to the optimization of the radiolabeling parameters, the distribution ratio, *D*, of ^111^In in a mixture of chloroform and HEPES buffer containing tropolone had been determined. The obtained distribution ratio is 4.8 ± 0.5 indicating that in the presence of tropolone, indium has a clear preference for the organic phase, due, most likely, to the formation of an In-tropolone complex having lipophilic character. The results for *D* at pHs other than 7.4, given in Additional file 1: Table S1, show a comparable *D* values for pH 4.5 to 7.4 and a lower *D* for pH 8.5, i.e., 2.9 ± 0.2.

**Fig. 3:**
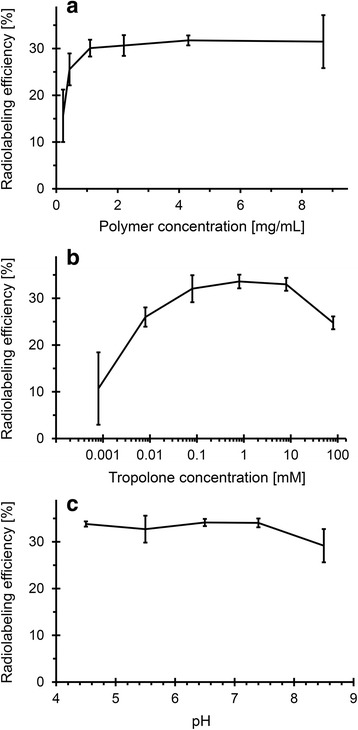
The radiolabeling efficiency. As function of **a** PS-b-PEO concentration (0.8 mM tropolone and pH 7.4), **b** tropolone concentration (4.3 mg/mL PS-b-PEO and pH 7.4), and **c** pH (4.3 mg/mL PS-b-PEO and 0.8 mM tropolone). In all experiments, 50 kBq ^111^In has been used. The *uncertainty bars* are the standard deviations (*N* = 3)

The radiolabeling efficiency increases with the polymer up to a value of 1.1 mg/mL, after which the encapsulated percentage remains constant, reaching a plateau at about 30 % (Fig. 3a). At a block copolymer concentration of 4.3 mg/mL, there is an excess of micelles compared to the number of ^111^In atoms (see Additional file 2 for the calculations), which implies that the labeling efficiency should be independent of the amount of indium. Indeed, the labeling efficiency appears to be the same up to the equivalent activities of 500 MBq (see Additional file 3: Figure S1).

The optimal tropolone concentration has been determined to be between 0.1 and 10 mM (Fig. 3b). At the lowest tested tropolone concentrations (0.8 μM), more than 50 % of ^111^In has been found to stick to the glass vial or the stirrer, while above 10-mM free tropolone is expected to compete with ^111^In-tropolone leading to a decrease in loading.

Figure 3c shows the effect of the pH on the radiolabeling efficiency, revealing that even at pH 4.5, the labeling efficiency is still 34 ± 1 %, and it remains constant up to pH of 7.4. Only at higher pH values, >8, the efficiency slightly decreases to 29 ± 4 %.

As an alternative to the passive loading method, the radiolabeling of preformed micelles, referred to as ‘active loading’ has also been tested since it has previously been successfully applied in the radiolabeling of liposomes and polymersomes [15]. In active loading, the ^111^In-tropolone complex in chloroform is added to the preformed micelles to transport the ^111^In into the core of the micelles. The labeling efficiency of this method has been shown to be somewhat lower than the passive loading, 22 ± 3 % versus up to 32 ± 2 %.

### Retention of ^111^In

**Fig. 4:**
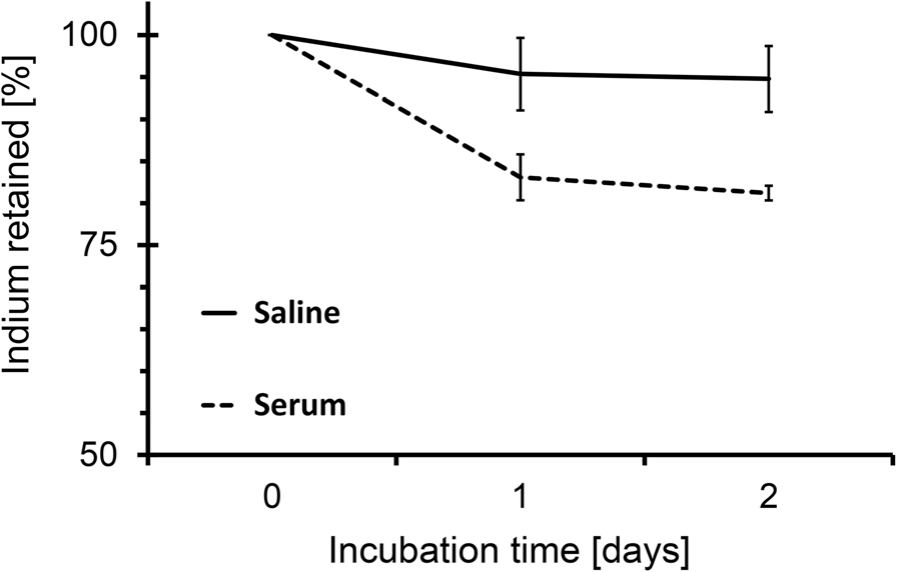
Retention of ^111^In in micelles as function of time. Polymer concentration 4.3 mg/mL, tropolone concentration 0.8 μM, and ^111^In activity 50 kBq per sample. The uncertainty bars are the standard deviations (*N* = 3)

The loss of radiolabel has been assessed in both phosphate-buffered saline solution and in mouse serum (Fig. 4). In saline, the micelles are stable having 95 ± 4 % of the ^111^In still encapsulated in the micelles after 2 days of incubation. The retention of ^111^In in serum is 83 ± 3 % and 81 ± 1 % after 1 and 2 days, respectively. In all samples, the same trend is observed: a fast release rate in 24 h followed by a negligible loss in the next 24 h.

### In vivo evaluation

**Fig. 5:**
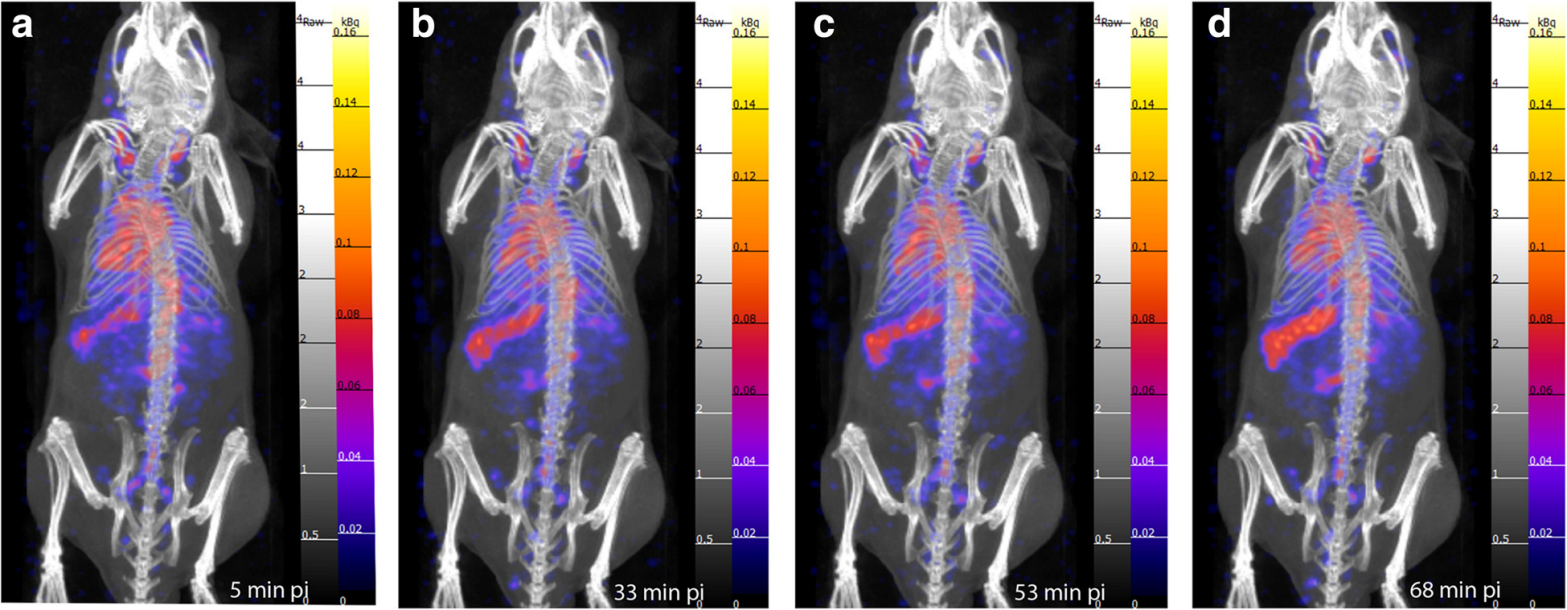
Dynamic SPECT/CT scans. Obtained after administration of 1 mg of ^111^In-PS-b-PEO micelles containing 22 MBq. **a** 5 min pi. **b** 33 min pi. **c** 53 min pi. **d** 68 min pi

**Fig. 6:**
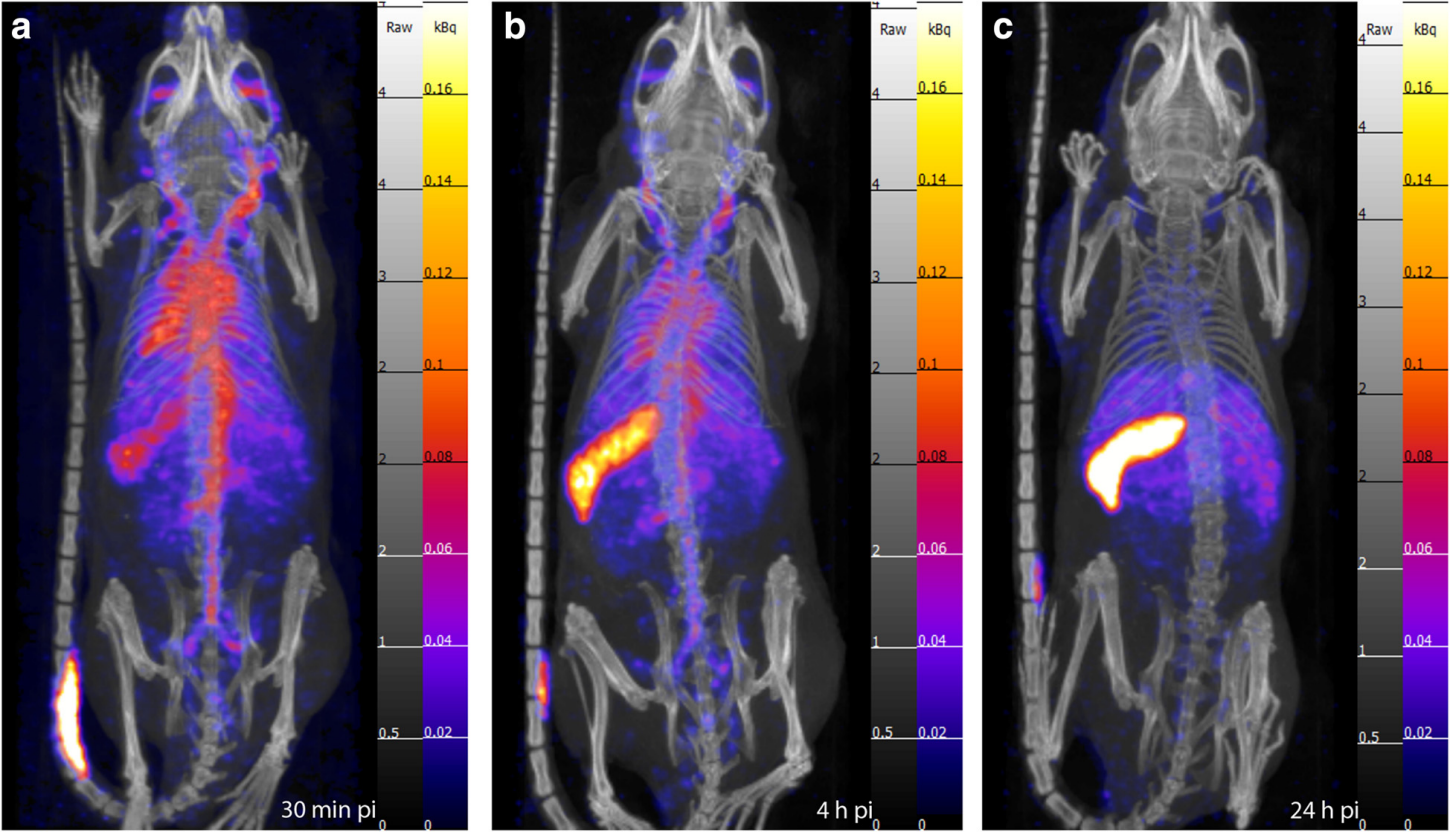
SPECT/CT static scans. Obtained at time points after the injection of 1 mg of ^111^In-PS-b-PEO micelles (22 MBq). **a** 0–30 min pi: circulation and uptake in the spleen. (**b**) 4 h pi: circulation still visible, although reduced in respect to **a. c** 24 h pi: circulation is not visible anymore, while most of the activity is in the spleen and in the liver

Dynamic SPECT/CT images in the first 68 min pi show that most of the activity is still in circulation (Fig. 5). Up to 4 h pi circulation is clearly detectable in the scans, making the major blood vessels and the heart chambers visible (Figs. 5, 6a, b), while at 24 h, the SPECT data suggests that the micelles have primarily accumulated in the spleen and liver and are not found in the blood (Fig. 6b, c).

**Fig. 7:**
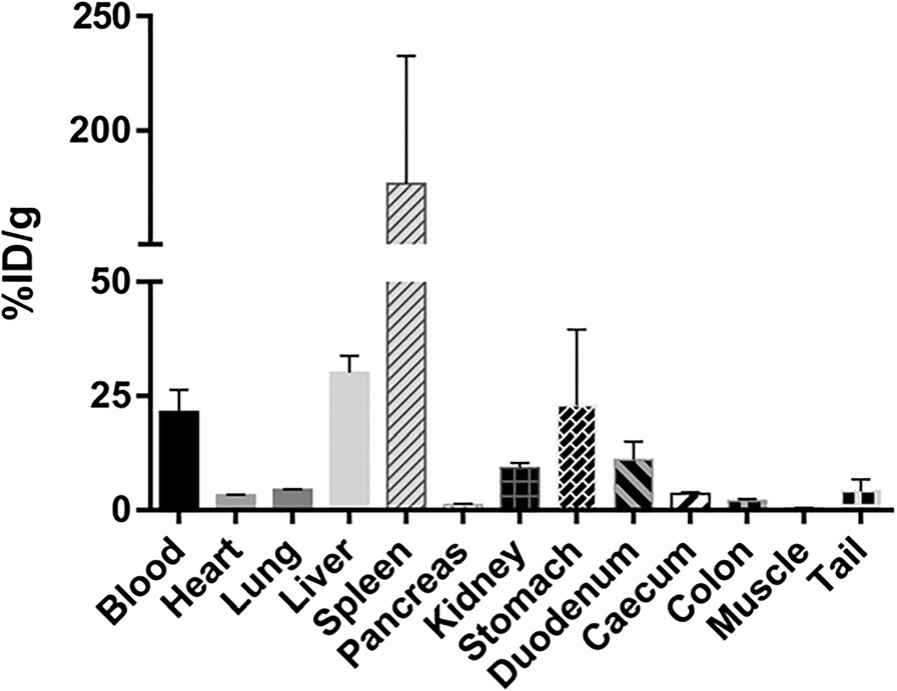
Biodistribution results 24 h pi. *Bars* represent the average values of the calculated % ID/g ± standard deviations (*N* = 3)

Whole-body retention of activity at 24 h pi has been estimated by measuring radioactivity levels in the extracted blood and the cadavers of euthanized animals, which show that more than 85 % of the injected activity has been retained in the body after 24 h. Ex vivo biodistribution analysis 24 h pi, expressed as a percentage of injected dose per gram of tissue (% ID/g), confirmed the observation made using SPECT/CT imaging, i.e., elevated spleen (176 ± 97 % ID/g) and liver uptake (30 ± 6 % ID/g) (Fig. 7). Surprisingly, although circulation is not detectable via nuclear imaging at 24 h pi, the blood samples reveal relatively high percentage of activity (21 ± 8 % ID/g), indicating clearly that the micelles are still in circulation at this time point. The stomach also showed relatively high uptake being on average around 22.96 % ID/g.

### Ex vivo autoradiography

**Fig. 8:**
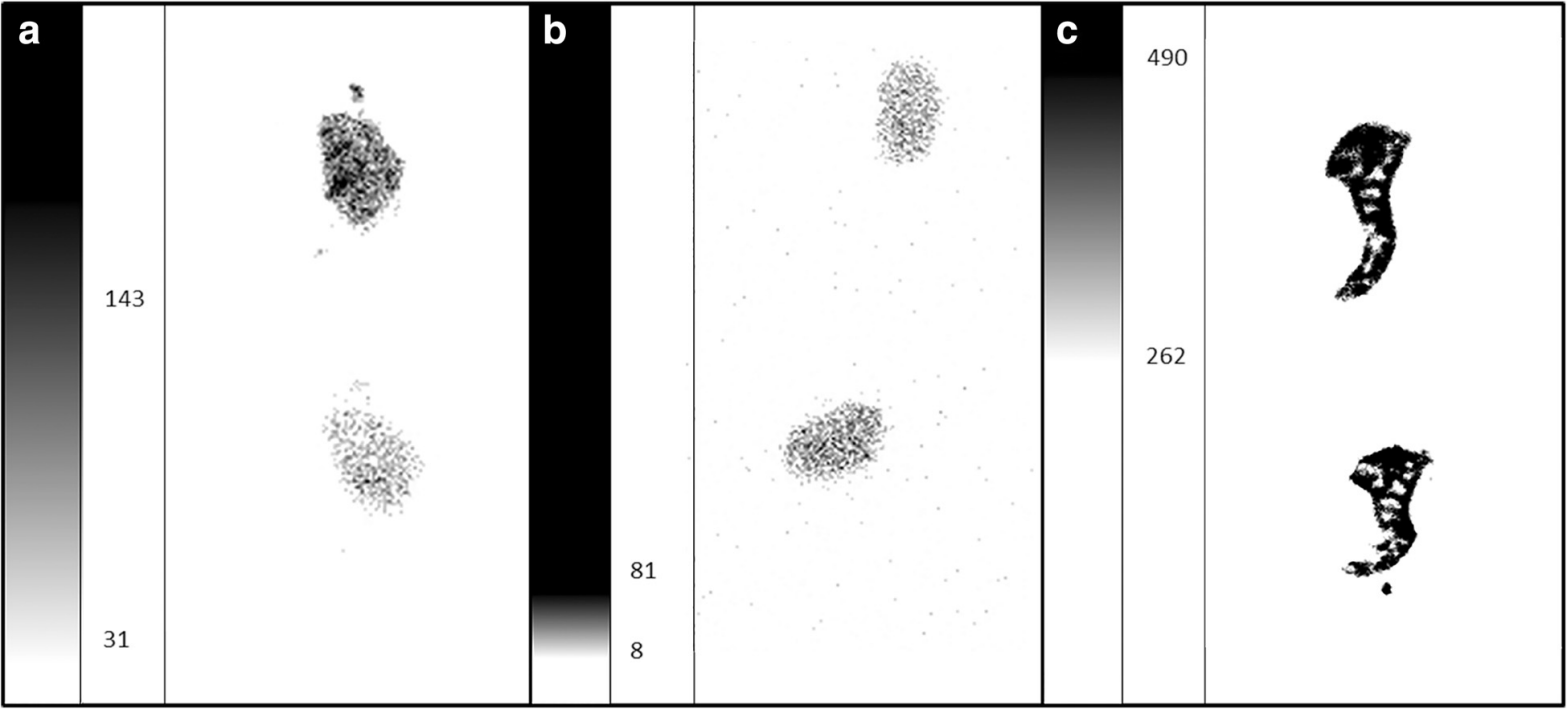
Autoradiogram showing the distribution of ^111^In-PS-b-PEO micelles in the liver, kidney and spleen 24 h pi. Two sections per organ are shown. The intensity scale is expressed in Digital Light Unit per surface (DLU/mm^2^) and adjusted per organ for optimal representation. **a** Liver (min 31; max 143). **b** Kidney (min 8; max 81). **c** Spleen (min 262; max 490)

Ex vivo autoradiography has been carried out in the sections of the spleen, liver, and kidney. Accumulation in the liver and the kidney tissue appears to be homogeneous. In contrast, the spleen has non-homogenous staining pattern having areas with elevated uptake and smaller zones with lower retention. The observed staining pattern coincides with the anatomical architecture of the spleen with red pulp and white pulp zones, indicating high accumulation in the red pulp and low accumulation in the white pulp (Fig. 8).

## Discussion

The aim of this study was to develop a simple and fast radiolabeling method for PS-b-PEO micelles which does not interfere with the intrinsic properties of micelles, allowing their evaluation in vivo using SPECT. Radiolabeling of polymeric carriers is typically carried out by the conjugation of a chelating molecule on their outer surface which has two main disadvantages: the properties of the nano-entities are altered and therefore possibly their behavior, and radiolabel integrity in vivo is often compromised [16]. For instance, recently, it has been shown that polymersomes composed of the same block copolymer and having the same size exhibit different biodistribution and loss of radiolabel when radiolabeling is on the outer surface versus enclosure in the aqueous cavity of the vesicles. The introduction of a chelating agent (i.e., DTPA) in this case resulted in somewhat negatively charged micelles which led to significantly higher liver uptake than when the PEG chains were not modified, preserving their neutrality [16]. The potential of polymeric micelles as carriers for therapeutic agents, especially when envisioning drug delivery applications, should, hence, be assessed without changing their inherent qualities. In this study, we make use of the physical characteristics of the block copolymer, PS-b-PEO (i.e., high *T*_g_ and extreme hydrophobicity) to encapsulate radionuclides in the micellar core, providing a novel and facile radiolabeling method that does not require chemical modifications, keeps the PEO corona intact, and protects the radiolabel from the biological environment.

The synthesis of the polymeric micelles is based on the spontaneous self-assembly of amphiphilic block copolymers into micellar structures due to formation of interfacial instabilities in chloroform-in-water emulsion droplets [17]. When the PS-b-PEO block copolymers dispersed in chloroform are emulsified in water, they distribute at the (water-chloroform) interface in order to reduce the entropy of the system, which eventually leads to the formation of micelles as the chloroform evaporates. The mobility of the PS-b-PEO unimers in the micellar core is virtually non-existent due to the glassy behavior of polystyrene, leading to kinetically trapped structures having exceptionally high stability [18]. This micellization process can be used to entrap lipophilic entities, such as in this case tropolone, which are present in the chloroform solution. Tropolone, being a ligand often used in radiolabeling, can be applied to passively bring a radio-metal to the micellar core, which is initially chloroform-rich, provided that the tropolone-metal complex has affinity for chloroform. The final evaporation of chloroform from the micellar structures results then in radiolabeled micelles. In this work, tropolone is complexed with indium (^111^In) and their distribution ratio has been determined to be above 1, showing a tendency to accumulate in the chloroform phase. Since radiolabeling is in this case a passive process, the radiolabeling efficiency will be limited by the distribution ratio and the amount of chloroform present in the micellar core during micellization, which naturally will depend on the number and size of the micelles. This inherent limitation, however, makes the radiolabeling procedure highly reproducible, since there is only a slight dependence on the different radiolabeling parameters such as polymer, tropolone, and indium concentration, and surprisingly, also pH, as long as sufficient micelles are formed. For instance, the efficiency remains nearly the same for tropolone concentrations ranging from 0.1 to 10 mM. Only at higher amounts a decrease is seen which is explained by the fact that if there is an excess of free tropolone, i.e., percentage-wise less ^111^In-labeled tropolone will be encapsulated, assuming that both species behave in a similar way (i.e., have a similar *D* value). The lack of dependence on indium concentration is logical since there is always a large excess of tropolone and polymer in the studied range, which allows encapsulating sufficient amount of activity at radiolabeling efficiency of 30 %. One of the parameters expected to affect the radiolabeling efficiency is the pH, simply based on the pKa value of tropolone and the speciation of indium. In the tested pH range of 4.5 to 8.5, the radiolabeling efficiency remains the same between pH 4.5 and 7.4 and only at pH 8.5 a small decline is observed. The same trend is observed for the distribution ratio, which is constant between pH 4.5 and 7.4 and is lower at pH 8.5. This decrease in the distribution ratio and in the labeling efficiency can be explained by the fact that at alkaline pH $\mathrm{In}{\left(\mathrm{O}\mathrm{H}\right)}_4^{-}$ is formed which is unable to complex with the negatively charged tropolone (see Additional file 4: Figure S2 for the speciation chart). The anticipated reduction of labeling efficiency at lower pH (<6.8) based on previous studies and the pKa value of tropolone (i.e., pKa 6.9) have not been observed. The structure of tropolone suggests that it will be partially situated at the water-chloroform interface and as such might have a different pKa value, enabling it to still extract indium even at somewhat acidic pH. This assumption also seems to be supported by the indium retention studies in serum, which reveal initial fast loss of radiolabel in the first 24 h followed by nearly no loss of any indium in the following 24 h. Such a loss profile can be ascribed to the distribution of In-tropolone in the micelles, i.e., it is very likely that some ^111^In-tropolone complexes will be situated at the interface between the PEO corona and the PS core and will be much more prone to be taken from the micelles by scavenging proteins. Nevertheless, the loss of radiolabel is still limited (<19 %) and acceptable.

The radiolabeling procedure described in this manuscript is based on some general chemical principles and may be applicable to other block polymers exhibiting similar behavior as PEO-b-PS, i.e., having very slow exchange kinetics. In addition, this procedure would also be suitable for (simultaneous) encapsulation of a variety of radionuclides, provided that they are capable of complexing with tropolone or similarly behaving lipophilic ligands.

The success of any micellar formulation in systemic application is mainly dependent on the capacity of carriers to accumulate in diseased tissue with limited retention in healthy organs. The blood residence time has been identified as one of the most crucial aspect in achieving high tumor uptake [19], which in turn depends on the capability of the micelles to escape the body clearance mechanisms. Carriers of the size of PS-b-PEO micelles are usually cleared by macrophages in the liver and spleen, often also referred to as the mononuclear phagocyte system (MPS). PS-b-PEO micelles show a substantial retention in MPS organs, in line with previous observations evaluating the in vivo behavior of other nanoparticles similar in size and composition [20, 21]. In this respect, the morphology of the micelles influences the circulation time and the presence of rod-like micelles in our case might affect the circulation time; however, as the number of elongated species is low, the effect on the average circulation time is considered to be negligible. A particular pattern of accumulation has been observed in the spleen confirming the possible uptake by the splenic resident macrophages as a result of unspecific filtration process [22]. This spleen uptake is in general higher when compared to other block copolymer micelles that have been investigated, which tend to exhibit more pronounced liver uptake [23]. This discrepancy in biodistribution can be due to differences in charge of the investigated systems, and as mentioned earlier, all other systems include chelates present on the surface of the micelles, which typically result in negatively charged micelles. In addition, the rigidity of the PS-b-PEO particles makes them more prone to spleen filtration. Apart from the blood, liver, and spleen, however, all the investigated organs show a low uptake profile (in average below 5 % ID/g) and little standard deviations indicative of a differential but homogeneous uptake in the various tissues. The surprisingly elevated stomach uptake is the result of one single animal, which explains as well the high standard deviation reported in the graph. This unexpected finding is probably not the result of tissue uptake/retention but could be ascribed to mice feeding behavior, i.e., daily coprophagy and social grooming [24]. Although we have not determined the half-live of the micelles, the measured retention in blood is at 24 h still above 20 % ID/g which indicates that a large percentage of the micelles are still in circulation, which is comparable to a number of studies on polymeric micelles [23]. Based on these results, we expect that these micelles will have long enough circulation time to ensure high tumor uptake, which should be further tested in tumor-bearing mice.

## Conclusions

We have developed a new, easy, fast, and robust method to produce and radiolabel spherical PS-b-PEO block copolymer micelles. The radiolabeling efficiency is sufficient to achieve micelle activities of up to several hundred megabecquerel. Ex vivo results show blood circulation even 24 h pi, with high accumulation in the spleen and liver. Overall, these results are promising for further investigation of radiolabeling polymeric micelles and for the assessment of their fate in vivo.

## Additional files

Additional file 1:
**Distribution ratio of indium-tropolone at pH 4.5 to 8.5.** In this file the experimentally determined distribution ratio of the indium tropolone complex for different pH values are given. Additional file 2:
**Calculation of the number ratio of** ^**111**^ **In nuclides and micelles.** In this file calculated values can be found which indicate how many ^111^In atoms are present in one micelle. Additional file 3:
**Effect of the indium activity on the radiolabeling efficiency.** In this file is shown how the radiolabeling efficiency is affected by the indium activity. Additional file 4:
**Speciation chart of indium over the full pH range.** In this file the relative abundance of the different indium species at pH 1 to 14 can be found.

## Abbreviations

DLS: dynamic light scattering
PEO: poly(ethylene oxide)
PET: positron emission tomography
PS: polystyrene
SPECT: single photon emission computed tomography
TEM: transmission electron microscopy
