# Supplementary material for: Radiolabeling polymeric micelles for in vivo evaluation: a novel, fast, and facile method
Source: EJNMMI Res. 2016 Feb 9;6:12. doi: 10.1186/s13550-016-0167-x (PMC4747947; doi:10.1186/s13550-016-0167-x)
Supplement: Additional file 1: — Distribution ratio of indium-tropolone at pH 4.5 to 8.5. In this file the experimentally determined distribution ratio of the indium tropolone complex for different pH values are given. [file 13550_2016_167_MOESM1_ESM.pdf]

ADDITIONAL INFORMATION

Distribution ratio of indium-tropolone at pH 4.5 to 8.5

Table a1. The distribution ratio (*D*) at pH 4.5 to 8.5, each value for D is an average and standard deviation of three measurements.

**pH**	***D***
4.5	4.7 ± 0.4
5.5	4.5 ± 0.5
6.5	4.8 ± 0.5
7.4	4.8 ± 0.5
8.5	2.9 ± 0.2
