# Supplementary material for: Radiolabeling polymeric micelles for in vivo evaluation: a novel, fast, and facile method
Source: EJNMMI Res. 2016 Feb 9;6:12. doi: 10.1186/s13550-016-0167-x (PMC4747947; doi:10.1186/s13550-016-0167-x)
Supplement: Additional file 2: — Calculation of the number ratio of 111 In nuclides and micelles. In this file calculated values can be found which indicate how many 111In atoms are present in one micelle. [file 13550_2016_167_MOESM2_ESM.pdf]

ADDITIONAL INFORMATION

Calculation of the number ratio of ^{111}In nuclides and micelles

For the calculation of the number of micelles we have used the aggregation number from the publication by Wilhelm et al.[25] For a PS-b-PEO block copolymer with a comparable size, i.e. for PS-b-PEO 11200-17500 an average aggregation number of 290 have been reported. At a PS-b-PEO 9500-18000 concentration of 1.1 mg/mL in a default volume 2.3 mL the number of micelles will be:

$$\frac{1.1 \times 10^{-3}\ g/mL \times 2.3\ mL}{(9500 + 18000)\ g/mole} \times \frac{6.022 \times 10^{23}\ n/mole}{290\ n/micelle} = 1.91 \times 10^{14}\ micelles$$

The number of ^{111}In nuclides is directly proportional to the activity and can be calculated with the decay constant, λ, which for ^{111}In is 2.86 x 10^{-6} $s^{-1.}$ At 50 MBq the number of ^{111}In atoms is:

$$N = \frac{A}{\lambda} = \frac{50 \times 10^{6} s^{-1}}{2.86 \times 10^{-6} s^{-1}} = 1.74 \times 10^{13}\ atoms$$

In this example there is about ten times excess of micelles compared to the number of ^{111}In atoms, meaning that part of the micelles will not contain any ^{111}In.
