# Supplementary material for: Radiolabeling polymeric micelles for in vivo evaluation: a novel, fast, and facile method
Source: EJNMMI Res. 2016 Feb 9;6:12. doi: 10.1186/s13550-016-0167-x (PMC4747947; doi:10.1186/s13550-016-0167-x)
Supplement: Additional file 3: — Effect of the indium activity on the radiolabeling efficiency. In this file is shown how the radiolabeling efficiency is affected by the indium activity. [file 13550_2016_167_MOESM3_ESM.pdf]

ADDITIONAL INFORMATION

Graphical representation of the effect of the indium activity on the radiolabeling efficiency

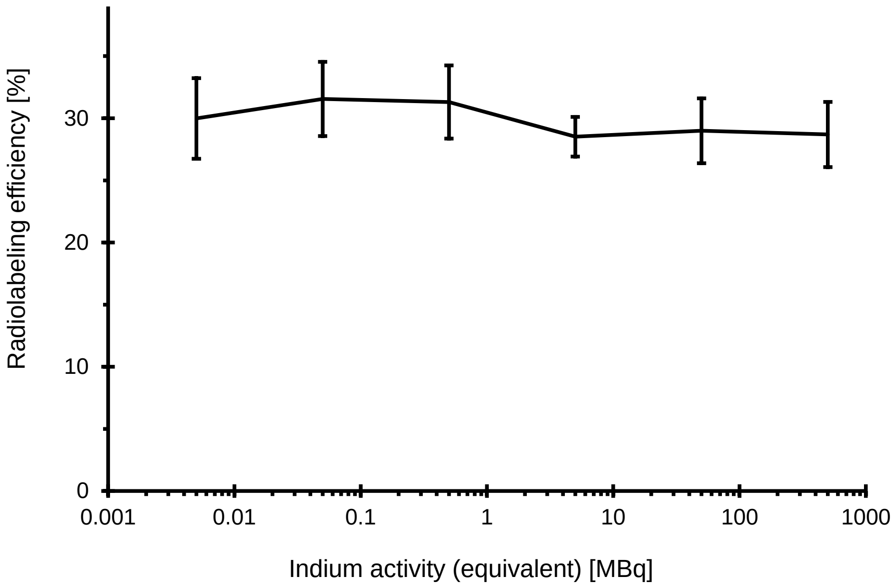

Figure a1. The labeling efficiency as function of the indium activity. The samples were prepared in HEPES buffer pH 7.4 with 0.8 mM tropolone and 50 kBq ^{111}In activity, with the exception of the lowest data point, for which 5 kBq was used, containing 4.3 mg/mL PS-b-PEO micelles. Increasing amounts of non-radioactive indium has been added equivalent to an ^{111}In activity up to 500 MBq. The uncertainty bars are the standard deviations (N=3).
