# Supplementary material for: Radiolabeling polymeric micelles for in vivo evaluation: a novel, fast, and facile method
Source: EJNMMI Res. 2016 Feb 9;6:12. doi: 10.1186/s13550-016-0167-x (PMC4747947; doi:10.1186/s13550-016-0167-x)
Supplement: Additional file 4: — Speciation chart of indium over the full pH range. In this file the relative abundance of the different indium species at pH 1 to 14 can be found. [file 13550_2016_167_MOESM4_ESM.pdf]

ADDITIONAL INFORMATION

Speciation chart of indium over the full pH range

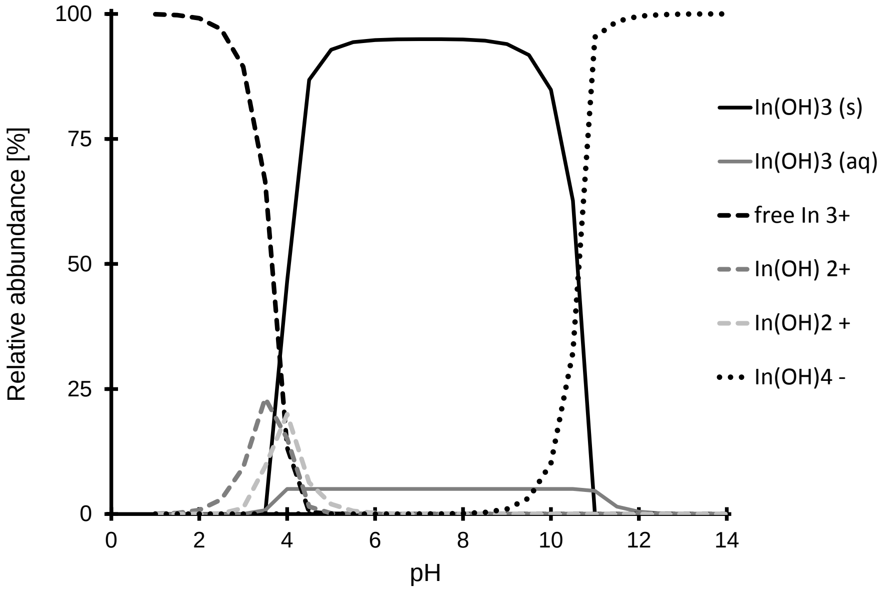

Figure a2. Speciation chart of indium at a concentration of 1 µM. The speciation chart is created with CHEAQS Next software version 2014.0.9.4. Input values for the concentration of both indium and chloride are $1x10^{-6}$ M, and for each pH the regarding H^+ input value has been set as free activity, the concentration of all other elements has been set to 0 M for this evaluation.
